# Editorial: Food, nutrition and microecological health

**DOI:** 10.3389/fnut.2023.1129273

**Published:** 2023-03-06

**Authors:** Xingbin Yang, Xin Liu, Guifang Tian, Hongbao Li

**Affiliations:** ^1^Shaanxi Engineering Laboratory for Food Green Processing and Safety Control, and Shaanxi Key Laboratory for Hazard Factors Assessment in Processing and Storage of Agricultural Products, College of Food Engineering and Nutritional Science, Shaanxi Normal University, Xi'an, China; ^2^Department of Epidemiology and Biostatistics, School of Public Health, Xi'an Jiaotong University, Xi'an, China; ^3^Department of Food Nutrition and Safety, College of Food Science and Technology, Hebei Agricultural University, Baoding, China; ^4^Department of Physiology and Pathophysiology, Xi'an Jiaotong University, Xi'an, China

**Keywords:** gut microbiota, dietary intakes, microecological homeostasis, bioactive ingredients, gastrointestinal health

Food nutrition and microecological status play important roles in maintaining physical and psychological health throughout the entire human life. Daily food intake provides us with nutrients, which are processed in the digestive tract and absorbed by intestinal epithelial cells (IECs). Some of the nutrients and the corresponding metabolites interact with the gut microbiota, a key factor for the homeostasis of gastrointestinal microenvironment, and are assimilated by the human body for the energy to run cellular processes and for the building blocks of proteins, lipids, nucleic acids, and carbohydrates. Therefore, understanding bioactive components from food and their influence on microecological health *via* the crosstalk with gut microbiota has attracted worldwide attention in recent years and has become one of the hotspots in food nutrition, biomedicine, and metabonomics research. Given the importance of this, we have launched this Research Topic named “*Food, Nutrition and Microecological Health*.” The topic is aimed at addressing the effects of specific food intake and specific food-derived compounds on microbiota alterations in human body, and their potential role in optimizing human health status, preventing and improving non-communicable diseases such as cardiac-cerebral vascular malfunctions, immunosuppression, hyperlipidemia, and hyperglycemia, hopefully to present some examples of “food dependent healthy microecological system.”

After 8 months' call for contributions, 26 papers of pertinence to the topic were accepted as publications on *Frontiers in Nutrition*, including 24 research articles and two comprehensive reviews. All the reported scientific discoveries addressed the gist of the topic from multiple aspects, ranging from bioactive natural products to toxic chemicals, with the purpose to elucidate the mechanisms of the network where food, nutrition, and microecological health are intensively interlacing. These contributions have provided new evidence on food-related changes in microbiota and the ensuing malfunctions and diseases, which will further promote the scope of precision nutrition and related studies. Here, we selected several papers with outstanding scientific discoveries, in hope of sharing the viewpoints with vast audience from the perspective of microecological health.

Nucleotides (NTs), the building blocks constituting DNA and RNA that account for more than 30% dry weight of human cells, are once considered as the conditional nutrients specially for infants, elderly, and patients in recovery. However, a new discovery has been made by Ding et al. that dietary NTs are able to slow down the senescence progress of SAMP8 mouse by promoting the constitution of gut microbiota. They added NTs to the basal diet of mice, and analyzed the intestinal microbiome and metabolites after 10 months when aging was observed. The findings showed that the supplementation of NTs enhanced the diversity of gut microbiota, while inhibited the bad bacteria (e.g., *Verrucomicrobia*) and increased the good (e.g., *Lactobacillus*) ones. As a result, the risk of intestinal microbiota dysbiosis that usually happens to senescent mice was reduced by the intervention of dietary supplementation. Further metabonomics studies revealed that the metabolism processes of histidine, vitamin B6, and linoleic acid were involved in the NT-mediated microecological improvement. Those biological events were closely related to the vitality of *Lactobacillus casei*, a well-characterized probiotic, and its antagonism against intestinal pathogens. Finally, they concluded that NTs is an overlooked prebiotic, which is able to regulate the imbalance of gut microbiota caused by aging.

A clinical study led by Wallingford et al. discussed the effects of oligosaccharides on healthy infants born in the USA and Honduras. The project was conducted jointly by researchers from Denmark and the US, who added the 2-fucosyllactose (2′ FL), a type of oligosaccharides rich in breast milk, to the infant formula to check if there was any changes in the intestinal microbiome of the infants. They found that, although the addition of 2′ FL failed to promote the growth of the infants, no adversary effects were observed on the formula-fed infants supplemented with level physiologic of 2′ FL. The oligosaccharides mediated modest changes in gut microbiome of the formula-fed infants, toward the direction of the infants fed with breast milk. *Bifidobacterium*, which has genes that allow intracellular metabolism of 2′ FL, was selectively enriched with the addition of 2′ FL, supporting the prebiotic property of 2′FL when used as a supplementation in infant formula. This study elucidated the nutritional functions of 2′ FL in infants lacking of breastfeeding, shedding the light on the importance of exogenous and endogenous provision of oligosaccharides that could be used as prebiotics.

Another clinical study by Yu et al. including researchers coming from several institutions across China dissected the low-carbohydrate diet-induced weight loss from the perspective of microecological nutrition. They applied 16S rRNA and ITS rRNA sequencing techniques to analyze the changes of gut bacteria and fungi of overweight and obese adults before and after being subjected to the low-carbohydrate dietary intervention (e.g., Atkins diet). More than 90% of the participants achieved weight loss of at least 5% over the course of the diet. The diversity of gut bacteria and fungi increased after a weight loss of 5% and kept stable thereafter, featured by depletion of *Lachnoclostridium* and *Ruminococcus* from Firmicutes phylum and increasing of *Parabacteroides* and *Bacteroides* from Bacteroidetes phylum. The inter-kingdom analysis found an intensive covariation between gut fungi and bacteria, more than half of them associated with weight loss. The study discovered the links between fungi and bacteria in the diet-caused weight loss, confirming the fact that gut microbiota is a key player in the development of dietary-related metabolic disorders such as obesity.

*Schisandra chinensis*, a berry fruit native to far east region including the territories of China, Japan, Korea, and Russia, has long been used as tea or prescription of decoctions in traditional Chinese medicine. In alcohol-caused liver diseases featuring with liver steatosis and fibrosis, alcoholic hepatitis (AH) is of high incidence and accounts for more than 50% of short-term mortality. Xiang et al. characterized the components of *Schisandra chinensis* extract (SCE) using UHPLC-QE-MS, and assayed their hepatoprotective effects on AH mice. They found that SCE significantly ameliorated inflammation and oxidative stress of AH mice, along with fortifying the intestinal barrier function by improving gut microbiota and the corresponding metabolites. Specifically, the abundance of *Lactobacillus plantarum* and *Bifidobacterium breve*, serving as the producers of short-chain fatty acids (SCFAs) were increased by the administrations of SCE both *in vitro* and *in vivo*. Meanwhile, *Escherichia-Shigella*, which was markedly enriched in AH mice, was inhibited by SCE, partially contributing to the mitigation of hepatic inflammation, lipid accumulation, and intestinal dysfunction. This study suggested that *S. chinensis* might be an effective dietary supplement for prevention and treatment of alcohol-caused liver diseases. Another investigation on the alcohol-related liver disease by Zhang et al. was also selected under the current topic in terms of microecological health. They found that folic acid (FA), also known as vitamin B9, is able to mitigate alcohol-induced liver injury *via* gut-liver axis. FA accelerated lipid deposition, and inhibited alcohol-caused inflammation as evidenced by the decreased the levels of LPS (Lipopolysaccharide), ALT (Alanine aminotransferase), AST (Aspartate transaminase), and TG (Triacylglycerol) in the serum of mice. Besides, Verrucomicrobia, Lachnospiraceae_NK4A136_group, and Akkermansia, which significantly decreased in the mice exposed to the alcohol consumption, were increased when FA was applied. Further correlation analysis showed that the levels of the inflammation-inducing cytokines in liver were highly related to the alteration of gut bacteria that were responsive to the FA supplementation. Bacteroidota, Firmicutes, and unclassified_Lachnospiraceae mainly accounted for the FA-mediated liver restoration, suggesting the pivotal role of gut-liver axis in the progress of alcohol-induced liver damage.

Apart from gut-liver axis, gut-brain axis is also an emerging hotspot for researchers dealing with micronutrition and psychological health. Two papers that fall into this category were enrolled by the current topic. One of them is coming from Lu et al., who contributed a paper titled “The antidepressant effect of deoiled sunflower seeds on chronic unpredictable mild stress in mice through regulation of microbiota-gut-brain axis”. In their study, the deoiled sunflower seeds (DSFS) which are rich in tryptophan, a precursor of serotonin with antidepressant potential, were used as the supplementation to the chow diet of the mice. The results showed that the elevated plasma corticosterone and the reduced hippocampal serotonin, two indexes for the depression-related symptoms, were recovered by the DSFS diet. Besides, DSFS significantly elevated the abundance of the bacteria that were inversely associated with depressive behaviors. Some of them, such as *R. flavefaciens, C. scindens*, and *O. massiiensis* were positively related to the production of indoleacetaldehyde, contributing to the delayed consumption of L-tryptophan in the microenvironment of gut. The other work discussing the gut-brain axis in this topic was contributed by Huang et al. from China. They found that *P. histicola*, an emerging probiotic by recent studies, was able to ameliorate the depression state of the ovariectomized mice by improving the structure of intestinal microbiota. *P. histicola* significantly increased the abundance of gut microbiota, especially *Lactobacillus* and *Akkermansia*. Meanwhile, the cohoused mice were observed with better emotional state and neutral structure compared with the ovariectomized mice, suggesting the estrogen deficiency-induced depression was related to gut-brain axis. Further in-depth investigations showed that *P. histicola* helped repair intestinal leakage and promoted the expression of neurotrophic factors (BDNF and Ki-67) for hippocampal neurogenesis. The two studies have provided new insights of using nutrients or probiotics to relieve psychiatric disorders without resorting to the chemotherapies that are often accompanied by unpredictable side effects.

Tu et al. from the US investigated whether a black raspberry (BRB)-rich diet could ameliorate the inflammatory bowel disease in mice. Using a mouse model of DSS-induced intestinal inflammation, they found that the intervention of BRB was able to delay and even reverse the inflammation progression in the small intestine. The anti-inflammatory effects were closely related to the modulation of the gut microbiota, especially to those bacteria that could produce AHR ligands in response to the BRB dietary intervention. This study demonstrated again that nutrients from natural products have great potentials to relieve inflammations by restoring the imbalanced gut microecology. Apart from the study on inflammatory bowel disease, two independent research groups both discussed the protective effects of food ingredients on renal damage. Wang et al. found that Oat β glucan, one of the major bioactive substances from oat, was able to modulate the composition of intestinal flora by regulating several metabolism processes, in turn alleviate the inflammatory response, and finally contribute to the postponed progression of nephropathy caused by diabetes. Zhu et al. from Fudan University of China used α-ketoacid as a supplement to low protein diet to assay the effects on the nephrectomized mice. The results showed that the low protein supplemented with α-ketoacid diet (LKD) could delay the adverse effects caused by the renal failure. The mice fed with LKD were observed with increased α-diversity and decreased F/B ratio of gut microbiota compared with that of mice in the control group. Further multi-omics analysis revealed that specific metabolites involving in multiple biosyntheses (such as glycerophospholipid, purine and vitamin B6) were associated with the amelioration effects of LKD on renal damage, which could be ascribed to the regulation of affecting the gut microbiome and fecal metabolic profiles. Results from the three studies put forward a notion of using food-derived interventions to exert prophylactic and therapeutic effects on chronic diseases *via* affecting intestinal flora and gut homeostasis.

Green banana flour (GBF) is a powder made of unripe bananas that are peeled, chopped, dried, and then ground. GBF has been used as a cheaper alternative to wheat flour in Africa, Jamaica, and some countries in South America. Recent studies show that GBF can be used as a gluten-free replacement for wheat flour, as well as a source of resistant starch, which has been promoted as a prebiotic to exert beneficial effects on gut microbiota. Li et al. found that, the addition of GFB in daily diet contributes to the restorations of gut microbiota and colonic barrier integrity of mice subjected to antibiotic perturbation. The mice were exposed to certain amount of ciprofloxacin and metronidazole once a day for 2 weeks, followed by GBF intervention for another 2 weeks. Compared with the natural recovery, GBF decreased the antibiotic-induced gut permeability by increasing the secretion of mucin, and promoted the growth of *Bacteroidales S24-7, Lachnospiraceae, Bacteroidaceae*, and *Porphyromonadaceae* that have beneficial effects on gastrointestinal homeostasis. The current study demonstrated that GBF could be used to alleviate the negative effects of antibiotics on gut microenvironment, paving the way of using GBF as a functional food ingredient to shield the gut microbiota and the intestinal barrier from being perturbed by antibiotics. Cui et al. from China contributed a paper investigating the effects of whole-grain diet (WGD) on gut microbiota of elderly individuals. Although they did not observe any changes in the α-diversity of the gut microbiota, WGD affected the abundance of Verrucomicrobia and Firmicutes. The influence of WGD varied significantly in terms of weight and gender, yet the decreased Firmicutes/Bacteroidetes ratio, which is conducive to the homeostasis of the gut microbiota, was observed. Hence, they concluded that WG could improve the microbial composition and promote the growth of beneficial microbes, which may be beneficial to the elderly individuals.

In summary, the studies above-mentioned provided enormous amount of emerging findings on the cross-talk between food components and gut microbe, contributing to beneficial effects on human health and guidelines and further research perspective in terms of precise nutrition. Granted, bad effects from both plant- and animal-derived food should also be discussed and investigated by taking microecological nutrition into consideration. The total 26 papers are certainly not able to cover all the aspects of the current topic. More and more good works with robustness and novelty are expected from researchers worldwide, some of which might be designed and conducted in the following angles:

a. Interactions between specific diet and gut microbiota and the corresponding effects on cardiac-cerebral vascular health, immune activation, arthrosis, hyperlipidemia, hyperglycemia, and hypertension.

b. Bioactive food compounds and their derivatives over the course of digestion, and their functions on gut microbiota and related metabolic processes.

c. Regional speciality food, the particular functional compounds inside and the interplays with gut microbiota.

d. Bioactive substances in food delivery systems, targeting specific intestinal bacteria and the in-depth molecular mechanism.

## Author contributions

XY: organizing, writing, and editing. XL, GT, and HL: writing and revising. All authors contributed to the article and approved the submitted version.

